# Acute Respiratory Tract Infections among Hospitalized Palestinian Patients (2011–2016): A Retrospective Study

**DOI:** 10.1155/2021/5643134

**Published:** 2021-05-03

**Authors:** Rania Abu Seir, Wafa' Njoum, Rawan Najajrah, Dania Najjar, Mariam Ashour, Bassam Asakra, Nahla Samman, Osama Najjar

**Affiliations:** ^1^Department of Medical Laboratory Sciences, Al-Quds University, Jerusalem, State of Palestine; ^2^Faculty of Medicine, Al-Quds University, Jerusalem, State of Palestine; ^3^Allied Health Professions, Palestinian Ministry of Health, Ramallah, State of Palestine

## Abstract

Respiratory tract infections (RTIs) are a major public health concern. This study aims to investigate the profiles and epidemiological characteristics of acute RTIs and respiratory pathogens in Palestinian hospitalized patients. Clinical samples from hospitalized patients with symptoms of acute RTIs admitted between January 2011 and December 2016 were referred to the Palestinian Central Public Health Laboratory (PHCL) to identify the causative pathogen. Patients' demographic information and the results of the molecular identification were retrieved from the electronic database at the PHCL. A total of 15413 patients with acute RTIs were hospitalized during the study period. The causal agent was identified only in 28.7% of the patients. Overall, influenza viruses were the most common cause of RTIs among hospitalized Palestinian patients in the West Bank. Children and elderlies were the most affected with RTIs. The elderly population (≥60 years old) had the highest rates. After influenza A virus, respiratory syncytial virus (RSV), and *Bordetella pertussis* (*B. pertussis*) were the most common causes of acute RTIs among hospitalized Palestinian patients. Children showed the highest hospitalization rates for RSV, *B. pertussis,* adenovirus, enterovirus, and *Streptococcus pneumoniae.* On the other hand, elderlies had the highest rates of influenza. Outbreaks of RTIs occurred mainly during winter (between December and March). The resurgence of *B. pertussis* in spite of vaccination is alarming and requires further investigation.

## 1. Introduction

Respiratory tract infections (RTIs) are considered to be the most common infectious diseases worldwide and the second leading cause of death among children under five years old [1, 2]. In Palestine, infectious diseases cause less than 10% of all deaths; respiratory diseases (ICD10 code: J00–J99.9) cause 70% of those deaths with a mortality rate of 17.0 per 100,000 population during 2016, being the sixth most common cause of death [3].

The etiological agents of respiratory diseases include a wide range of respiratory viruses and bacteria. They present with a spectrum of symptoms that include fever, cough, malaise, and chest pain [4]. Rapid interventions are necessary as these infections could result in either mild illness or could lead to severe complications, hospitalization, and death [4, 5]. Identification of the causative agent of respiratory diseases based on signs and symptoms alone is not reliable [6]. Therefore, understanding the epidemiology of RTIs and identification of the patterns and etiologies are critical for successful treatment and prevention programs [7]. This is very important as 14.9% of the Palestinian population are children <5 years of age [3].

During the last few decades, a shift in the burden of disease from communicable to noncommunicable diseases has been noticed in many developing countries [8]. Nevertheless, infectious diseases continue to be a major cause of morbidity and mortality among Palestinians, especially children under 5 years old [3]. Respiratory diseases can be prevented through public health measures [9]. In developing countries, *B. pertussis*, enteroviruses, influenza viruses, RSV, adenoviruses, and *S. pneumoniae* are considered to be the main causes of RTIs resulting in 4-5 million annual deaths among children only [10]. The purpose of the current study was to investigate the profiles and epidemiological characteristics of acute RTIs and respiratory pathogens in hospitalized patients in the West Bank, Palestine.

## 2. Materials and Methods

A retrospective study was conducted during the period from January 2011 to December 2016 among hospitalized Palestinian patients. Demographic data and laboratory results were retrieved from the health information system at the Palestinian Central Public Health Laboratory (PHCL) by the research team through the electronic system. Demographic data included gender, age, place of residence, and date of sampling (hospitalization).

PHCL is under the administration of the Ministry of Health (MOH), the main healthcare provider in Palestine. This advanced laboratory operates at the national level and receives samples from all over the West Bank for testing.

Samples of hospitalized patients with symptoms of acute RTIs are transferred from all MOH hospitals in the West Bank and delivered to the PHCL for testing by polymerase chain reaction (PCR). These samples include nasopharyngeal aspirates, nasopharyngeal swabs, oropharyngeal swabs, sputum, blood, and bronchoalveolar lavage fluid and are collected routinely by qualified medical personnel in the hospitals and sent to PHCL to confirm clinical diagnosis. The samples are tested for the suspected pathogen. In case of a positive laboratory result for the suspected pathogen, the case was considered confirmed; otherwise, it was considered suspected. Laboratory testing is available for microorganisms including *Bordetella pertussis* (*B. pertussis*), enterovirus, influenza A virus, influenza B virus, respiratory syncytial virus (RSV), adenovirus, and *Streptococcus pneumoniae* (*S. pneumoniae*).

This study was undertaken using data from the PHCL with the approval of MOH. The data were previously anonymized, and no private information was collected as part of this study. Therefore, no approval from an ethics committee or informed consent from patients was required for this study.

Data were analyzed using IBM SPSS statistics version 20.0. Descriptive statistics were done in the form of means, frequencies, percentages, and ranges of the variables. Categorical variables were evaluated by the chi‐square test. All *p* values less than 0.05 were considered statistically significant. Population data for the calculation of rates were obtained from the Palestinian Central Bureau of Statistics (PCBS) 2011–2016. Rates were calculated using Microsoft Office Excel 2010.

## 3. Results

A total of 15413 Palestinian patients were hospitalized between 2011 and 2016 with acute RTIs.  Table 1 shows the demographic characteristics of the cases. Acute RTIs were equally common among males and females. The mean age of cases was 32.8 years (range 0–107). Of the cases, 31.6% were less than ten years old.

The highest incidence rates of acute RTIs were observed among children less than 10 years old and elderlies (≥60 years old). The mean incidence rate of hospitalization for acute RTIs in the West Bank during the study period was 91.4 per 10^5^ population (range 34–149 per 10^5^). During the six years of the study, hospitalization rates for acute RTIs increased, but a drop was recorded during 2014. Overall, hospitalization rates were highest in the northern governorates of the West Bank.

During the study period, the causal agent was only identified in 28.7% of the cases. The highest detection rate was for RSV followed by influenza A viruses (Table 2). When we compared the number of cases of each organism between males and females, we did not detect significant differences (*p* value = 0.299) (Table 2).

**Figure 1:**
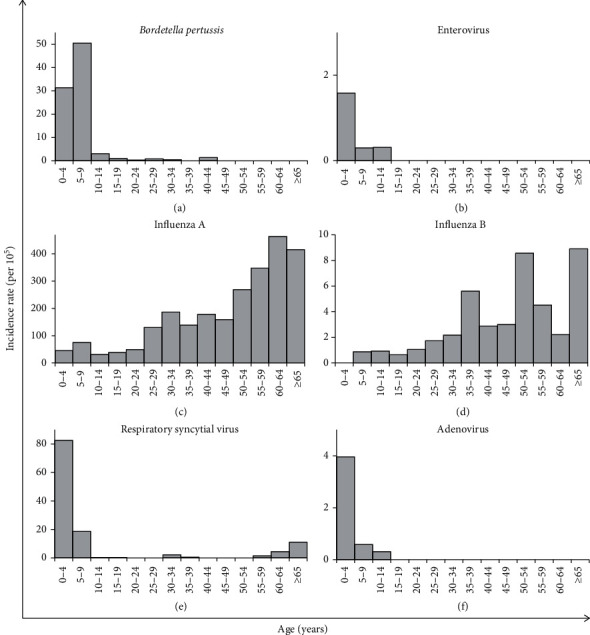
Incidence rates of major causes of respiratory tract infection-related hospitalization (2011–2016) stratified by age, West Bank. (a) *B. pertussis*; (b) enterovirus; (c) influenza A; (d) influenza B; (e) RSV; (f) adenovirus.

Influenza A was the major cause of acute RTIs among hospitalized patients with confirmed laboratory results (Table 2). Between 2011 and 2013, the hospitalization rate increased from 4.3 to 30.0 per 10^5^ population, and between 2015 and 2016, from 26.0 to 39.3 per 10^5^ population. A sharp drop between the two periods (during 2014) was noticed (Table 3). Hospitalization rates of influenza A virus increased with age, especially after the age of 60 years (Figure 1). The highest number of cases was recorded during cold months, December and January (Table 4). There were no cases of avian flu (H5N1) recorded during the period between 2011 and 2016, but swine flu (influenza A (H1N1) pdm09) was relatively common with a total of 1373 confirmed cases. Hospitalization rates of swine flu were highest during 2016 (IR = 13.7 per 10^5^) and 2013 (IR = 13.4 per 10^5^) (Tables 2 and 3). The highest rates were seen in northern governorates (Table 5). As for influenza B, hospitalization rates were relatively low, being highest during 2011 (Table 3).

The second major cause of hospitalization for RTIs was RSV (Table 2). The highest RSV hospitalization rates were among children <5 years old, followed by children between 5 and 9 years old (Figure 1). Although RSV hospitalization rates were highest among children, elderlies (≥60 years old) also had increased rates. The rates of RSV were highest during 2016 and 2014, respectively (Table 3). Further, the central governorates recorded threefold higher rates as compared to northern and southern ones (Table 5). Like influenza A, most cases occurred during December and January (Table 4).

Regarding pertussis cases, they were mainly children and the highest rates were seen among those between 5 and 9 years old (Figure 1). During 2012, the hospitalization rate was almost five times higher compared to the other years. Since 2013, a gradual increase in hospitalization rates was observed (Table 3). About half the number of cases occurred during spring (March-June) (Table 4) and the central region showed the highest rate while the lowest rates were observed in the south (Table 5).

## 4. Discussion

This study investigated the most common causes of acute RTIs among hospitalized patients in the West Bank. The data obtained through this study are population-based and therefore useful for predicting disease burden, in addition to planning for vaccine research and control strategies. During the study period, 15413 cases of severe acute RTI cases were hospitalized at the Palestinian hospitals in the West Bank. Hospitalization rates increased during the study period from 2011 to 2016. In summary, this study showed that the highest rates of hospitalization due to RTIs were observed among children less than 10 years old and elderlies (≥60 years old). Viruses including influenza and RSV were the main causes of RTIs among hospitalized patients in the West Bank. Among children, RSV was the major cause of RTIs, while among the other age groups influenza was the most common cause of RTIs.

Similar patterns were reported worldwide [4]. Data from the surrounding countries are limited and offer no comparisons to our study. For instance, a study conducted in Amman, the capital of Jordan, between September 2002 and March 2004, reported RSV to be the most common viral cause of respiratory tract infections among hospitalized children <2 years old accounting for 46.3% of diagnosed respiratory viruses [11]. On the other hand, in Beirut, Lebanon, between October 2013 and September 2014, human rhinovirus (23%), RSV (19%), human bocavirus (15%), human metapneumovirus (10%), and human adenovirus (10%) were respectively reported as the most common causes of RTIs among hospitalized children aged 16 years old or less [12], while in the Egyptian Delta, between June 23, 2009, and December 31, 2013, influenza was the major viral cause of RTIs (13.9%) and RSV was reported as the major cause among children <1 year old (∼40%) [13].

Our findings show that the largest percentage of hospitalized patients was children less than ten years old, but the highest hospitalization rates were among the older population. This is simply explained by the fact that the Palestinian population is a young population with one quarter the population being less than ten years old [14].

Although the highest number of cases overall was associated with influenza A, most cases of RSV, adenoviruses, *B. pertussis*, and enteroviruses occurred in children less than ten years old. These organisms were previously reported to be most common among children [15–18]. Further, RSV had the highest incidence among children less than 5 years old, but another increase in RSV hospitalization rates was observed among elderlies (≥60 years old). RSV is reported as the leading cause of RTIs among children worldwide [4]. Similar findings were reported in neighboring areas [11, 13, 19–21]. Susceptibility to RSV infection decreases with age as a result of maturation of the immune system [22]. On the other hand, in line with our observations, a population-based surveillance of RSV infection in the Nile Delta Region in Egypt (2011–2012) showed that although the highest hospitalization rates for RSV were among children less than 5 years old, another peak in hospitalization rates was observed among population ≥50 years old [23]. Another study conducted in Thailand reported a similar increase in hospitalization rates for RSV among those 65 years of age or more [24]. The role of RSV in the elderly is not yet understood, but it is clear that its burden is significant [25].

*B. pertussis* is a vaccine-preventable disease that used to be considered a universal infection among children less than 5 years old. The infection was reported to kill one in ten infected children in the United States during the 1920s [26]. Whole-cell pertussis vaccine was available since the 1940s, and during the nineties, it was replaced by acellular vaccines. Studies have shown that immunity against pertussis is not lifelong [26]. In Palestine, vaccination against *B. pertussis* is a part of the Expanded Program on Immunization and is given in combination with vaccines against tetanus, diphtheria, and Hib at the ages of 2, 4, and 6 months, in addition to a booster dose at 18 months. Our study showed that *B. pertussis* is still considered a major cause of morbidity in the West Bank. Outbreaks of the disease have been reported worldwide regardless of the high vaccine coverage [26, 27]. Studies showed that the waning immunity results in a peak in the incidence of pertussis among school-age children, and the infection spreads from these subjects to infants or not-fully vaccinated young children [27]. In addition, changes in the circulating strains of the bacterium should be considered [26, 27]. Our findings showed similar patterns. The severity of infection among infants is the highest [26]. In this study, we only included hospitalized patients, reflecting severe cases of the disease, and excluding mild, nonhospitalized cases who are probably older (adolescents and young adults).

In contrast to RSV and pertussis, influenza cases occurred mostly in the elderly population, a pattern of influenza that has been previously demonstrated [28, 29]. Similar findings were reported in the Egyptian Delta, where influenza was the major cause of acute RTIs and was most common among individuals 65 years old or more [13].

In Palestine, the seasonal influenza vaccine is not part of the national immunization program (NIP), but we have seasonal influenza vaccination policies in place. Influenza vaccination recommended the influenza vaccine for people with chronic illnesses, pregnant women, residents of long-term care facilities, pilgrims, and healthcare workers. The vaccine is available through both the public and the private sectors, but there are no data regarding vaccine coverage [30].

Furthermore, in our study, the observed pattern of influenza A virus through the study years showed a drop in the rate of hospitalized cases of influenza A during 2014 followed by a gradual increase in 2015 and 2016. This could be related to the varying severity of the circulating strains from one year to another [1]. Another possible reason for this drop is the matching between vaccine composition and circulating strains which in turn increases vaccine efficacy [31]. In addition, our findings showed a relatively high number of H1N1 cases. The Eastern Mediterranean Region (EMR) was affected by the worldwide increase of pandemic spread of H1N1 [32]. On the other hand, no cases of avian flu (H5N1) were found in our study. In fact, since the start of the H5N1 epidemic in the region, no human cases of H5N1 have been recorded in Palestine [32]. In the absence of an influenza surveillance system, the data remain insufficient to be confident about the circulating patterns.

*S. pneumoniae* is a major cause of morbidity and mortality. The introduction of pneumococcal conjugate vaccines (PCVs) has reduced invasive pneumococcal disease in all age groups, yet the burden remains high, mainly due to the emergence of other serotypes not included in the vaccines [33]. In Palestine, PCV10 was introduced in 2011 as part of the pediatric national immunization program in a 2 + 1 schedule. The laboratory diagnosis of invasive pneumococcal disease still relies on culture-based methods. In addition, recent developments occurred with antigen detection assays and nucleic acid amplification tests. Yet, invasive pneumococcal disease caused by *S. pneumoniae* can be difficult to confirm microbiologically [34]. Our study showed that, during the period between 2011 and 2016, only a very small number of samples (*n* = 18) were tested for *S. pneumonia* and only 17% of these samples tested positive.

The use of antibiotics without a prescription is a common practice in our community; as a result, physicians can rarely obtain viable samples for testing, which could explain the low number of referred samples. Although PCR contributed significantly to the detection and diagnosis of invasive pneumococcal disease as it is faster and more sensitive in comparison with standard culture (which is slow and yields false negative as a result of antibiotic treatment prior to sampling), among hospitalized patients, the long time between the onset of symptoms and disease progression contributes to poor sensitivity of detection of *S. pneumoniae* in nasopharyngeal swabs by PCR among hospitalized patients [34–38].

The highest incidence of RTIs was recorded during the cold season (December-March) throughout the study years. Several studies reported annual epidemics of respiratory diseases during the winter season in temperate climates such as Palestine [39–43]. Outbreaks of influenza during the rainy season were reported worldwide [1, 44]. Explanation of the seasonality of infections has been hard. One of the hypothesized explanations states that these seasonal outbreaks are a result of overcrowding indoors with the lack of proper ventilation during cold seasons [45, 46]. In addition, low temperature and dry conditions were found to be favorable conditions for pathogen transmission in animal studies, which is consistent with indoor state [45, 46]. A study in Bangladesh that investigated the number of influenza cases and weather factors showed that influenza A was associated with lower temperatures, relative humidity, sunlight duration, and rainfall [47]. The hospitalization rates from other organisms were very low during the study period. Therefore, we were unable to review patterns and trends accurately.

In summary, RTIs are still a public health concern, especially among children and elderly populations. Influenza viruses are the major cause of respiratory diseases among Palestinians. RSV is the most common cause of pediatric RTIs while influenza is the major cause among the elderly population. In addition, *B. pertussis* is still a common cause of RTIs among children regardless of the vaccination policies.

This study is the first to describe RTIs in Palestine and is one of the largest studies in the neighboring countries [11–13]. The study included all MOH hospitals in the West Bank, which is the main healthcare provider in Palestine. In addition, the study covered a period of six years, which was suitable to capture changes in the trends of the disease. Regardless, one of the limitations of this study was including only hospitalized patients in MOH hospitals without considering outpatients and patients in other hospitals; inclusion of these cases could have provided a more comprehensive and representative view on the patterns of RTIs. Furthermore, some common respiratory tract infections were not examined here as they are not part of the diagnostic tests such as human rhinovirus, human bocavirus, and human coronavirus. Moreover, clinical characteristics of the cases and disease outcomes were not considered and we did not include a control group in our study. Finally, coinfection with two or more pathogens, which could be an indication of increased risk for clinical outcome, is another missing entity in this study.

## 5. Conclusions

We compared the spectrum, seasonality, and age distribution of common causes of RTIs among Palestinians in the West Bank. Our data showed that viral agents caused the majority of respiratory diseases. Further surveillance and follow-up on the epidemiology of these diseases are recommended. In most cases of RTIs, the causal agent was undetermined; accurate and rapid diagnosis of the etiological agents are important to select the most effective treatment and avoid complications of the disease that could result in prolonged hospitalization and even death. Furthermore, interventions and policies that promote judicious antibiotic use should be implemented. Lastly, epidemiologic investigation for pertussis should be launched to identify factors and interventions to control these outbreaks of cases and vaccination campaigns against seasonal flu should target the elderly population.

## Figures and Tables

**Table 1:** Frequency and incidence rates of hospitalized patients with RTIs in the West Bank (2011–2016).

Variable	Category	Frequency *N* = 15413 *N* (%)	Incidence rate^a^ (per 10^5^)
Gender	Male	7974 (52.0)	567.1
	Female	7349 (48.0)	539.7
Age (years)	0–9	4427 (31.6)	615.2
	10–19	944 (6.7)	147.8
	20–29	1560 (11.1)	302.8
	30–39	1817 (13.0)	527.7
	40–49	1478 (10.6)	484.0
	50–59	1447 (10.3)	906.2
	≥60	2337 (16.7)	1732.1
Region^b^	North	6535 (42.4)	601.4
	Center	3713 (24.1)	467.1
	South	5165 (33.5)	582.7
Year	2011	880 (5.7)	34.1
	2012	2150 (13.9)	80.1
	2013	3110 (20.2)	112.9
	2014	1402 (9.1)	50.2
	2015	3511 (22.8)	122.7
	2016	4360 (28.3)	148.5

^a^IR: incidence rate (hospitalized patients per 10^5^ population). Population was calculated as the average overall population in the West Bank between 2011 and 2016 according to PCBS data. ^b^Region: North: Nablus, Tubas, Jenin, Tulkarem, Salfit, and Qalqiliya Governorates; Center: Ramallah and Al-Bireh, Jerusalem and Jericho, and Al-Aghwar Governorates; South: Hebron and Bethlehem Governorates.

**Table 2:** Frequency and incidence rates of confirmed respiratory tract infection-related hospitalization stratified by gender.

Organism	Suspected (*n* = 15413)	Confirmed (*n* = 4422)		Male		Female	
	*N* (%)	*N* (%)	IR^a^	*N* (%)	IR^a^	*N* (%)	IR^a^
*B. pertussis*	1403 (9.1)	324 (7.3)	11.7	160 (7.5)	11.4	153 (6.8)	11.2
Influenza A	11593 (75.2)	3555 (80.4)	128.4	1695 (79.4)	120.5	1848 (81.7)	135.7
Influenza A (H1N1)	5379 (34.9)	1373 (31.0)	49.6	667 (31.3)	47.4	702 (31.0)	51.6
Influenza B	699 (4.5)	61 (1.4)	2.2	29 (1.4)	2.1	32 (1.4)	2.3
RSV	1271 (8.2)	449 (10.2)	16.2	233 (10.9)	16.6	213 (9.4)	15.6
Enterovirus	139 (0.9)	9 (0.2)	0.3	4 (0.2)	0.3	5 (0.2)	0.4
Adenovirus	290 (1.9)	21 (0.5)	0.8	10 (0.5)	0.7	11 (0.5)	0.8
*S. pneumonia*	18 (0.1)	3 (0.1)	0.1	3 (0.1)	0.2	0 (0.0)	0.0

^**a**^IR: incidence rate (hospitalized patients per 10^5^ population). Population was calculated as the average population in the West Bank between 2011 and 2016 according to PCBS data.

**Table 3:** Frequency and incidence rates of respiratory tract infection-related hospitalization stratified by year.

Organism	Year					
	2011	2012	2013	2014	2015	2016
	*N* (IR^a^)	*N* (IR^a^)	*N* (IR^a^)	*N* (IR^a^)	*N* (IR^a^)	*N* (IR^a^)
*B. pertussis*	36 (1.4)	158 (5.9)	16 (0.6)	21 (0.8)	42 (1.5)	51 (1.7)
Influenza A	111 (4.3)	622 (23.2)	827 (30.0)	97 (3.5)	744 (26.0)	1154 (39.3)
Influenza B	28 (1.1)	3 (0.1)	10 (0.4)	11 (0.4)	7 (0.2)	2 (0.1)
RSV	5 (0.2)	46 (1.7)	61 (2.2)	107 (3.8)	87 (3.0)	143 (4.9)
Enterovirus	0 (0.0)	1 (0.0)	0 (0.0)	1 (0.0)	4 (0.1)	3 (0.1)
Adenovirus	0 (0.0)	1 (0.0)	0 (0.0)	2 (0.1)	6 (0.2)	12 (0.4)
*S. pneumoniae*	0 (0.0)	0 (0.0)	0 (0.0)	1 (0.0)	0 (0.0)	2 (0.1)

^**a**^IR: incidence rate (hospitalized patients per 10^5^ population). Population was calculated as the average annual population in the West Bank between 2011 and 2016 according to PCBS data.

**Table 4:** Frequency of confirmed respiratory tract infection-related hospitalization stratified by the month of occurrences.

Organism	Month (*N*)											
	Jan	Feb	March	Apr	May	June	July	Aug	Sep	Oct	Nov	Dec
*B. pertussis*	20	19	35	39	39	41	29	20	14	25	24	19
Influenza A	1203	581	482	187	71	12	5	5	12	19	112	866
Influenza B	18	7	14	9	8	2	0	0	0	0	1	2
RSV	142	63	71	50	10	4	3	3	3	1	15	84
Enterovirus	0	0	0	0	0	0	3	1	2	1	1	1
Adenovirus	2	4	5	2	1	1	0	0	1	1	1	3
*S. pneumoniae*	0	0	0	0	0	0	0	0	0	0	2	1
All RTIs	3974	2464	2444	1349	727	239	167	189	228	333	773	2526

**Table 5:** Frequency and incidence rates of confirmed respiratory tract infection-related hospitalization stratified by region.

Organism	North^a^		Center^a^		South^a^	
	*N* (%)	IR^b^	*N* (%)	IR^b^	*N* (%)	IR^b^
*B. pertussis*	136 (42.0)	12.5	120 (37.0)	15.1	68 (21.0)	7.7
Influenza A	1695 (47.7)	156.0	592 (16.6)	74.5	1268 (35.7)	143.1
Influenza A (H1N1)	624 (45.5)	57.4	228 (16.6)	28.7	521 (37.9)	58.8
Influenza B	22 (36.0)	2.0	14 (23.0)	1.8	25 (41.0)	2.8
RSV	113 (25.2)	10.4	264 (58.8)	33.2	72 (16.0)	8.1
Enterovirus	6 (66.7)	0.6	2 (22.2)	0.3	1 (11.1)	0.1
Adenovirus	0 (0.0)	0.0	3 (14.3)	0.4	18 (85.7)	2.0
*S. pneumonia*	0 (0.0)	0.0	2 (66.7)	0.3	1 (33.3)	0.1

^a^Region: North: Nablus, Tubas, Jenin, Tulkarem, Salfit, and Qalqiliya Governorates; Center: Ramallah and Al-Bireh, Jerusalem and Jericho, and Al-Aghwar Governorates; South: Hebron and Bethlehem Governorates. ^b^IR: incidence rate (hospitalized patients per 10^5^ population). Population was calculated as the average overall population in the regions of the West Bank between 2011 and 2016 according to PCBS data.

## Data Availability

The data that support the findings of this study are available from the Palestinian Ministry of Health, but restrictions apply to the availability of these data, which were used under license for the current study, and so are not publicly available. Data are however available from the authors upon reasonable request and with permission of the Palestinian Ministry of Health.
